# Mitogenomic Insights Into the Population Structure and Demographic History of Tree Shrews ( *Tupaia belangeri* ) in China

**DOI:** 10.1002/ece3.74338

**Published:** 2026-09-10

**Authors:** Tongtong Gu, Lijuan Cao, Dongjie Liu, Chengyao Yang, Zixin Lin, Rongxiang Mu, Wanlong Zhu

**Affiliations:** ^1^ School of Life Sciences Yunnan Normal University Kunming China

**Keywords:** demographic history, genetic diversity, genetic structure, mitogenome, *Tupaia belangeri*

## Abstract

The northern tree shrew ( *Tupaia belangeri* ) exhibits significant morphological and geographical variations, but its evolutionary history and subspecies boundaries remain controversial. Here, we analyzed the complete mitochondrial genomes of 63 individuals, representing 12 populations in China to study phylogenetic relationships, genetic diversity, and population history. Phylogenetic analysis consistently restored four mitochondrial branches with strong geographic structures and significant differences. The three lineages correspond to geographically restricted subspecies (*T. b. tonquinia*, *T. b. modesta*, and *T. b. gaoligongensis*), while individuals assigned to several traditional subspecies cluster in a broad mainland lineage (*T. b. chinensis*, *T. b. yunalis*, and *T. b. yaoshanensis*). The divergence time estimate places the origin of the main lineage in the Miocene, consistent with major tectonic and geomorphological events. Demographic analysis revealed different population histories, including varying degrees of expansion in recent continental and island lineages, as well as the long‐term stability of *T. b. gaoligongensis*. Genetic diversity varied markedly among lineages, with the highest diversity observed in the *T. b. gaoligongensis* and the lowest diversity observed in the *T. b. modesta*. These findings demonstrate that landscape complexity and demographic history are key drivers of evolutionary diversification in *T. belangeri* , challenging classical morphology‐based subspecies classifications and underscoring the need for comprehensive sampling across both domestic and international ranges.

## Graphical abstract

Sample information and genetic landscape of the northern tree shrew.

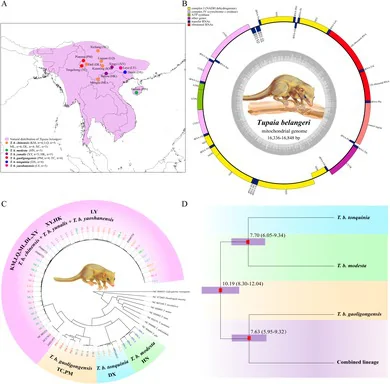

## 1 Introduction

The tree shrew is a small omnivorous mammal widely distributed in central South Asia and Southeast Asia, occupying terrestrial and arboreal ecological niches. Among them, the Northern treeshrew ( *Tupaia belangeri* ) is the only representative of the Scandentia order in China, representing an endemic species in the Eastern domain (Ruedi 1996). It has been included in the National List of Terrestrial Wildlife with Important Ecological, Scientific, and Social Value and Appendix II of the Convention on International Trade in Endangered Species of Wild Fauna and Flora (CITES) (Zheng et al. 2014). This species is mainly distributed in South Asia, Southeast Asia, and southwestern China, while the domestic population is distributed in Yunnan, Sichuan, Guizhou, Guangxi, and Hainan Island (Wang 1987; Roberts et al. 2011). Its scope spans different environments, including mountains, plateaus, and tropical islands (Sargis et al. 2016, 2018).

Tree shrews exhibit a predominantly facultative monogamous social system, wherein pairs maintain exclusive territories but may also engage in extra‐pair interactions. This social structure is primarily regulated through a repertoire of olfactory and acoustic signals (Schehka et al. 2007). Notably, other tree shrew species and Wells et al. (2008) have demonstrated female‐biased dispersal in a variety of small mammals (Munshi‐South 2008). Molecular studies have shown a close phylogenetic relationship between tree shrews and existing primates (Fan et al. 2019). Due to its small size, relatively large brain to body mass ratio, short reproductive cycle, short lifespan, and low maintenance cost, it has been recognized as a promising alternative to primates in biomedical research and is now considered an important experimental animal model after mice and macaques (Xu et al. 2012; Yu et al. 2016). However, most of the tree shrews used in the study still come from the wild or belong to genetically admixed populations, and the genetic background characteristics at the population level are still very poor. Although the key challenges of domestication and laboratory breeding of wild tree shrews have been addressed, the lack of comprehensive genetic data still hinders the establishment of standardized inbred lines.

There has been a longstanding debate over the subspecies classification of *T. belangeri* in China. Early assessments mainly relied on morphological characteristics. Allen (1938) recognized three subspecies: *T. b. chinensis* in western and central Yunnan, *T. b. yunalis* in southeastern Yunnan and Guangxi's Yaoshan region, and *T. b. modesta* on Hainan Island. Ellerman and Morrison‐Scott (1951) later proposed that only two subspecies occur in China, arguing that *T. b. yunalis* from southeastern Yunnan and *T. b. tonquinia* from northern Vietnam are likely conspecific with *T. b. modesta*. The most comprehensive and widely referenced revision based on geographical patterns and morphology was provided by Wang (1987), who recognized six subspecies: *T. b. chinensis*, *T. b. modesta*, *T. b. yunalis*, *T. b. gaoligongensis*, *T. b. tonquinia*, and *T. b. yaoshanensis* (Wang 1987). Corbet and Hill (1992) later supported only four of these, suggesting that *T. b. modesta* and *T. b. chinensis* represent the same subspecies. Helgen et al. (2005) further reduced the taxonomy, recognizing only *T. b. belangeri* and *T. b. chinensis*. More recently, genomic data have indicated that *T. b. chinensis*, *T. b. yunalis*, and *T. b. yaoshanensis* form a single lineage (Ren et al. 2023). These inconsistencies highlight the need for additional evidence to clarify the subspecies classification and distribution of *T. belangeri* in China.

The northern edge of the distribution of tree shrews has formed in southwestern China, and it is widely believed that tree shrews originated in tropical regions before moving northward (Roberts et al. 2011). The region was affected by major geological events, including the uplift of the Himalayas and the convergence of the China India Myanmar plate, which created a wide range of habitats and strong geographical barriers. These changes have influenced the formation of rivers, mountains, and forests, thereby affecting the animal dispersal routes. Therefore, the region is both a center of high biodiversity and an important pathway for species migration between the Palearctic and Oriental regions (Holt et al. 2013). These environmental and geological processes may have played an important role in shaping the population structure and evolutionary history of *T. belangeri* .

To better understand the complex genetic structure and evolutionary history of *T. belangeri* in China, we sequenced and assembled the complete mitogenome of individuals collected from main geographic populations. Using a complete mitochondrial genome instead of short mitochondrial fragments can provide more detailed genetic information and a clearer understanding of evolutionary relationships and population processes (Galtier et al. 2009; Morin et al. 2010; Cameron 2014). By combining mitogenomic data from 12 geographical populations, this study aims to explore genetic structure, genetic diversity, and demographic history, and provides useful insights for the scientific management and conservation of *T. belangeri* .

## 2 Materials and Methods

### 2.1 Sample Collection and DNA Extraction

We included 56 *T. belangeri* individuals from 12 populations in China, representing all currently divided subspecies according to Wang (1987). Wang's classification was originally based on a comprehensive assessment of geographical distribution and morphological characteristics. Accordingly, each specimen was assigned to a subspecies based on its documented collection locality and morphometric data, which aligns with the established distribution ranges of each subspecies: *T. b. chinensis* Kunming (KM), *n* = 4; Luquan (LQ), *n* = 5; Mengla (ML), *n* = 6; Dali (DL), *n* = 4; Xichang (XC), *n* = 5, *T. b. modesta* Hainan (HN), *n* = 5; *T. b. yunalis* Xingyi (XY), *n* = 5; Hekou (HK), *n* = 5; *T. b. gaoligongensis* Pianma (PM), *n* = 4; Tengchong (TC), *n* = 4; *T. b. tonquinia* Daxin (DX), *n* = 4; and *T. b. yaoshanensis* Leye (LY), *n* = 5. Those individuals were sourced from Ren et al. (2023), and we utilized the existing tissue samples for DNA extraction and subsequent analyses, ensuring no additional animal sacrifice was performed.

### 2.2 Sequencing, Assembly, and Annotation

Complete mitogenomes were sequenced using next‐generation sequencing (NGS) on an Illumina HiSeq 2000 platform (Biomarker Technologies, Beijing). The correct recognition rate of bases reaches 99.9%. Each Illumina HiSeq read was 150 bp. After obtaining the raw reads, we used FASTQ v1.0.1 (Chen et al. 2018) to remove adapters and low‐quality sequences, and about 0.8 Gb raw data were trimmed with default parameters. The clean paired reads were then used for mitogenome reconstruction using MITObim v1.9.1 (Hahn et al. 2013) and MitoFinder v1.4.2 (Allio et al. 2020) with default parameters and the mitogenome of *T. belangeri* (NC_002521) (Petrova et al. 2023) as the reference. The assembled mitogenomes were annotated using MitoFinder v1.4.2 (Allio et al. 2020). Circular maps of the mitogenomes were drawn using OGDRAW (https://chlorobox.mpimp‐golm.mpg.de/geseq.html) (Greiner et al. 2019). The 56 newly sequenced mitogenome sequences have been uploaded onto GenBank with the accession number PX872944–PX872999. Additionally, 14 published mitogenomes representing eight tree shrew species (Schmitz et al. 2000; Arnason et al. 2002; Xu et al. 2012; Kundu et al. 2022; Petrova et al. 2023) and an outgroup from Cynocephalidae (Arnason et al. 2002) were downloaded from NCBI and added to the mitogenome analyses.

### 2.3 Data Analysis

#### 2.3.1 Genetic Structure Analysis

A total of 71 sequences were used to reconstruct their phylogenetic relationships (Table S1). Two separate alignments were used to reconstruct Neighbor Joining (NJ), Maximum Likelihood (ML), and Bayesian inference (BI) phylogenetic trees, including the trimAl v1.5 (Capella‐Gutierrez et al. 2009) curated alignment and the concatenated protein‐coding genes alignment (PCGs).

In the first approach, all sequences were aligned using MAFFT v7.037 (Katoh et al. 2017), and then the complete mitogenome alignment was curated with trimAl v1.5 (Capella‐Gutierrez et al. 2009) to eliminate the divergent and poorly aligned regions of the alignment, forming 16,254 bps of the dataset. NJ tree was constructed using eGPS v1.0 (Yu et al. 2019) with the Kimura's two parameter model; ML tree was then calculated using IQTREE v1.6.12 (Kalyaanamoorthy et al. 2017), which automatically selects the best‐fit model, both 1000 bootstraps were performed to evaluate node support. The best nucleotide substitution model (GTR) for BI phylogenetic analyses was selected using ModelFinder integrated into IQTREE, based on the Bayesian information criterion (BIC) (Schwarz 1978). BI tree was carried out using MrBayes v3.2.6 (Ronquist et al. 2012) with the following requirements: two independent runs of 1 × 10^7^ generations were conducted with four independent Markov Chain Monte Carlo (MCMC) runs, including three heated chains and a cold chain, by sampling every 1000 generations. A consensus tree was obtained from all the trees after the initial 25% of trees from each MCMC run was discarded as burn‐in, with the chain convergence assumed after the average standard deviation of split frequencies fell below 0.01. The confidence value of each node of the BI tree was presented as the Bayesian posterior probability (BP).

In the second approach, the complete mitochondrial genes were extracted using PhyloSuite v2 (Zhao et al. 2025) and finally concatenated to make a single file containing all 13 PCGs with a total size of 11,362 bp. The protein‐coding sequence was also used to reconstruct the NJ and ML trees as described above. The BI tree was then carried out for 12,000,000 generations.

To further explore population structure, Arlequin v3.5 (Excoffier and Lischer 2010) was employed to perform an analysis of molecular variance (AMOVA) with 1000 permutations to determine the significance of genetic variation within and among populations. Mega v11.0.10 was employed to calculate pairwise genetic distances among haplotypes using the Kimura two‐parameter (K2P) model (Tamura et al. 2021). This model accounts for transition/transversion bias, which is appropriate for mitochondrial DNA sequences. The resulting distance matrix served as the basis for subsequent population differentiation analyses. DnaSP v5.10.01 (Librado and Rozas 2009) was employed to perform an analysis of population differentiation to determine the genetic variation among populations, providing a deeper understanding of the population structure.

#### 2.3.2 Genetic Diversity

Considering the consistent phylogenetic results of two datasets based on different methods, we conducted subsequent analysis using trimAl curated alignment with more informative loci. The number of haplotypes (*N*_h_) and haplotype diversity (H_*d*_) were calculated using DnaSP v5.10.01 (Librado and Rozas 2009). Haplotype diversity (H_*d*_), also known as gene diversity, was computed as $H_{d}=\frac{n}{n-1}\left(1-\sum_{i=1}^{k}p_{i}^{2}\right)$, where *n* is the sample size, *k* is the number of haplotypes, and $p_{i}$ is the frequency of $i^{\mathrm{th}}$ haplotype. This metric reflects the probability that two randomly selected haplotypes are different in the sample.

#### 2.3.3 Divergence Time Estimation

The estimation of divergence times was calculated using MDGUI module of PhyloSuite v2 with GTR model (Yang 2006; Huerta‐Cepas et al. 2016; Zhao et al. 2025). Three soft‐bound fossil calibration points were applied to constrain the analysis, acknowledging that while the fossil record provides minimum age constraints for clades, the actual most recent common ancestors (MRCAs) could be older; thus, soft bounds were employed to allow a small probability (typically 2.5%) of divergence times falling outside the specified intervals. The total tree height was considered 126‐67 MYA (Vries and Beck 2021), according to the fossil *Purgatorius mckeeveri* from the early Paleocene (Mantilla et al. 2021). The most recent common ancestor (MRCA) of Scandentia representatives was calibrated with 65–34 MYA, based on the fossil of *P. kylin* from the early Oligocene (Li and Ni 2016). The MRCA of *Tupaia* was calibrated with 28–18 MYA, based on the fossil of *T. miocenica* (Mein and Ginsburg 1997). Burn‐in was set at 1,000,000 iterations, and sampling was performed every 50 iterations until 10,000,000 samples had been gathered. Birth (*λ*), death (*μ*), and sampling (*ρ*) priors of *λ* = 1, *μ* = 1, and *ρ* = 0 were used. The transition/transversion rate ratio (kappa gamma), the shape parameter for rate heterogeneity between sites (alpha gamma), and the prior on rates (rgene gamma) were specified as (6, 2), (1, 1), and (1, 1.76), respectively. Consistency diagnosis of the two independent molecular dating runs was completed by MCMCTracer.

#### 2.3.4 Gene Flow and Demographic Analyses

We estimated gene flow among four genetic populations using Migrate‐n v3.6 (Beerli and Felsenstein 2001; Beerli 2005). The Markov chain Monte Carlo (MCMC) sampling included three long chains of 50,000 generations and ten short chains of 10,000 generations, with sampling performed every 200 generations. A burn‐in of 10,000 generations was applied to each chain. To ensure reliability, we performed five independent runs and used the average value across these runs as the final estimate of gene flow.

To reconstruct the population history of each population, neutrality test (Tajima's *D*) analyses was conducted using Arlequin v3.5 (Excoffier and Lischer 2010). Additionally, an Extended Bayesian Skyline plot (EBSP) was generated with BEAST v2.5 (Bouckaert et al. 2019) to assess changes in the effective population size (*N*_e_) of *T. belangeri* within each population, and the generation time and the mutation rate were separately set at 0.5 years and 2 × 10^−8^ per site per year in this study (Brown et al. 1979; Yu 2002). The HKY nucleotide substitution model (Hasegawa et al. 1985) was selected as the best‐fit model based on BIC in jModelTest v2.1.7 (Darriba et al. 2012) and applied in the EBSP analysis. Each MCMC simulation was run for 1,000,000,000 generations and sampled every 5000 generations. The convergence were estimated using the effective sample size (ESS) values (> 200 in all parameters) in Tracer v1.7.1 (http://beast.bio.ed.ac.uk/Tracer) (Rambaut et al. 2018). The EBSPs were visualized using an in‐house R script.

## 3 Results

### 3.1 Assembly the *T. belangeri*

**FIGURE 1:**
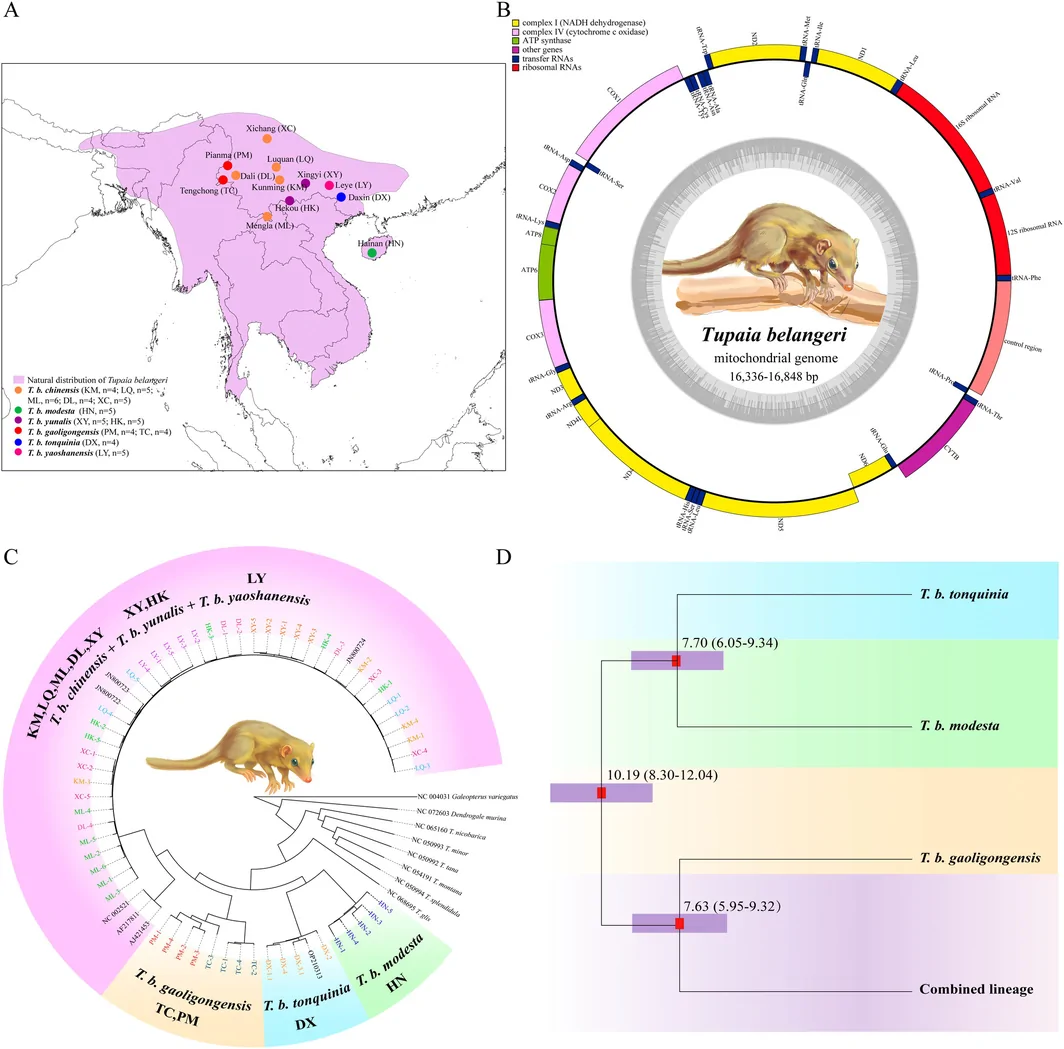
Sample information and genetic landscape. (A) The natural distribution and sampling information of *Tupaia belangeri* used in this study. (B) Circular maps of the mitogenome of *T. belangeri*. (C) Phylogenetic tree inferred from mitochondrial sequences (ML). The three tree‐building methods produced similar topology and here we show the ML tree. (D) Divergence time inferred from mitochondrial sequences. Interspecific divergence times are shown on the branches, including median values and their 95% HPDs (purple bars); The red boxes on the nodes indicate full bootstrap values at the nodes representing relationships within *T. belangeri* .

We sampled 56 individuals from 12 populations throughout Sichuan, Guizhou, Yunnan, Guangxi, and Hainan provinces (Figure 1A). The complete mitogenome of the *T. belangeri* was assembled and annotated in the current study. The total length of the mitogenome of *T. belangeri* ranges from 16,336 to 16,848 and encodes 37 genes, including two ribosomal RNAs (rRNAs) *12S* and *16S*, 22 transfer RNAs (tRNAs), 13 PCGs, and a *D‐loop* in the characteristic arrangement (Figure 1B). The percentage of GC pairs is about 40.5%. The nucleotide composition is A, C, G, and T of approximately 33.0%, 6.3%, 14.2%, and 26.5%, respectively. Nine genes (ND6 and eight tRNAs) were oriented in the reverse direction, whereas the others were transcribed in the forward direction. The minimum and maximum base coverages of the mitogenome were 320‐ and 4098‐fold, respectively, and the mean base coverage was 1513‐fold, and the standard deviation was 616 (Table S1).

### 3.2 Population Structure

Based on the phylogenetic analyses using trimAl curated alignment and PCGs databases, all 63 *T. belangeri* individuals (56 newly generated in this study and seven additional sequences obtained from GenBank) were resolved into four major clades with strong statistical support (ML and NJ, BS = 100%; BI, BP = 1.0) (Figure 1C and Figure S1). One branch consists solely of the *T. b. tonquinia* from DX, another is just the *T. b. modesta* from HN, and a third is the *T. b. gaoligongensis* from PM and TC. All the remaining individuals, representing *T. b. chinensis*, *T. b. yunalis*, and *T. b. yaoshanensis*, which come from a wider geographical range including KM, LQ, ML, DL, XC, XY, HK, and LY, all fall together into a single, fourth major branch, hereafter referred to as the ‘combined lineage’. The topology further revealed that the clade containing *T. b. tonquinia* and *T. b. modesta* was sister to a clade comprising *T. b. gaoligongensis* and the combined lineage. Genetic distances between these differentiated populations range from 0.093 to 0.118. Similarly, their corresponding FST values range from 0.792 to 0.948 (Table 1), presenting strong and significant genetic differentiation. Moreover, the genetic distance and differentiation level between *T. b. gaoligongensis* and the combined lineage are always the lowest. Based on the overall grouping of AMOVA, it was found that the variation of each grouping method mainly occurred among populations, with a variation ratio of 88.07%.

**TABLE 1:** Genetic distance (below diagonal) and differentiation degree (above diagonal) between four populations within *Tupaia belangeri*.

	Combined lineage	*T. b. tonquinia*	*T. b. modesta*	*T. b. gaoligongensis*
Combined lineage	0	0.926	0.926	0.792
*T. b. tonquinia*	0.115 ± 0.001	0	0.948	0.859
*T. b. modesta*	0.116 ± 0.001	0.106 ± 0.001	0	0.857
*T. b. gaoligongensis*	0.093 ± 0.002	0.118 ± 0.002	0.116 ± 0.001	0

### 3.3 Genetic Diversity

A total of 57 haplotypes were identified, including five from *T. b. tonquinia*, four from *T. b. modesta*, seven from *T. b. gaoligongensis*, and 41 from combined lineage (Table 2). In addition, the haplotype diversity was high across all subspecies, ranging from 0.900 ± 0.161 in *T. b. modesta* to 1.000 ± 0.126 in *T. b. tonquinia*. The nucleotide diversity was highest in *T. b. gaoligongensis* (0.025 ± 0.022, *p* < 0.05, Wilcox test), followed by the combined lineage (0.011 ± 0.004), while *T. b. tonquinia* (0.005 ± 0.001) and *T. b. modesta* (0.005 ± 0.001) showed relatively lower values.

**TABLE 2:** Genetic diversity of four populations within *Tupaia belangeri*.

Populations	Sample size	Haplotype diversity	Nucleotide diversity
Combined lineage	45	0.995 ± 0.006	0.011 ± 0.004
*T. b. modesta*	5	0.900 ± 0.161	0.005 ± 0.001
*T. b. tonquinia*	5	1.000 ± 0.126	0.005 ± 0.001
*T. b. gaoligongensis*	8	0.964 ± 0.077	0.025 ± 0.021

### 3.4 Divergence Dating

Two independent molecular dating analyses revealed consistent results and the separation time of the *Tupaia* genus is estimated as an average of 25.98 MYA and 95% highest posterior density (HPD) of 22.55–28.52 MYA (Figure 1D and Figure S2). The sister branches of *T. b. tonquinia* and *T. b. modesta* and the combined lineage and *T. b. gaoligongensis* differentiated 10.19 MYA (95% HPD: 8.30–12.04 MYA). The divergence between *T. b. tonquinia* and *T. b. modesta* occurred 7.70 MYA (95% HPD: 6.05–9.34 MYA). The combined lineage and *T. b. gaoligongensis* diverged 7.63 MYA (95% HPD: 5.95–9.32 MYA).

### 3.5 Demographic History and Gene Flow

**FIGURE 2:**
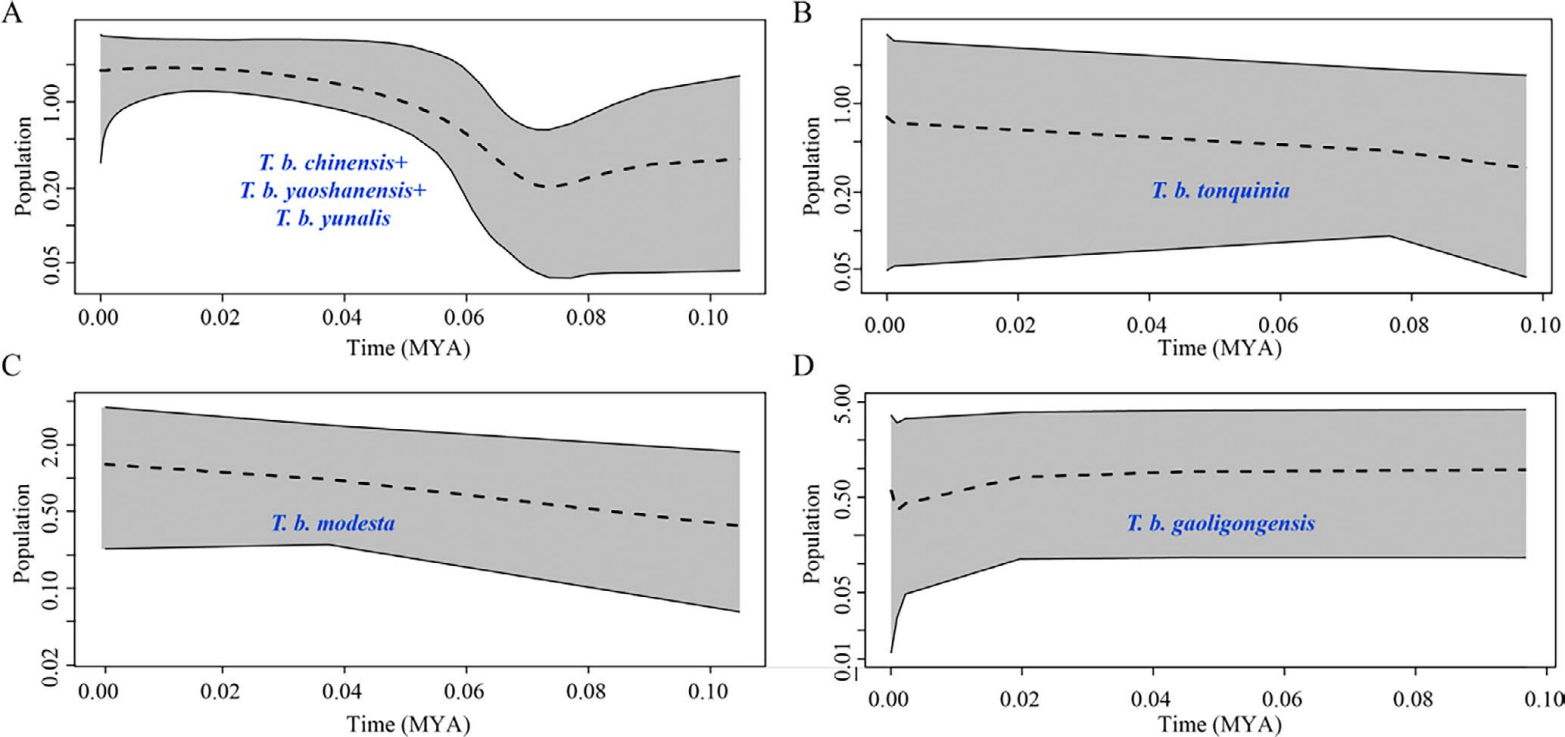
Extended Bayesian Skyline plot (EBSP) illustrating the demographic history of each subspecies. (A) Combined lineage, (B) *T. b. tonquinia*, (C) *T. b. modesta*, and (D) *T. b. gaoligongensis*. The dotted lines represent median estimates of effective population size over time, the shaded areas denote 95% confidence intervals surrounding the median estimates.

Among the four subspecies, only the combined lineage exhibited a significantly negative Tajima's *D* value, while the SSD and r statistics were not significant, indicating a history of population expansion (Table 3). The EBSP analysis further showed that following a period of decline in effective population size, the population experienced a significant increase (Figure 2A). Tajima's *D* values for *T. b. tonquinia* and *T. b. modesta* were also negative but not statistically significant (Table 3), and EBSP analyses indicated demographic growth in both subspecies (Figure 2B,C). In contrast, *T. b. gaoligongensis* was the only subspecies with a positive Tajima's *D* value (Table 3), and EBSP results suggested that after a prolonged period of demographic stability, this subspecies underwent a recent population decline (Figure 2D).

**TABLE 3:** The statistics of mismatch analyses, the test of selective neutrality, SSD and Raggedness Index.

Population	Mismatch			The test of selective neutrality	Sum of square deviations (SSD)	SSD's *p* value	Raggedness Index (RI)	RI's *p* value
	*τ*	*θ*_0_ before expansion	*θ*_1_ after expansion	Tajima's *D*				
*T. b. tonquinia*	56.000	72.000	3662.383	−0.490	0.089	0.060	0.200	0.950
*T. b. modesta*	70.750	21.600	7497.524	−0.458	0.183	0.000**	0.310	0.060
*T. b. gaoligongensis*	/	/	/	1.194	/	/	/	/
Combined lineage	45.605	14.732	687.122	−1.725**	0.002	0.770	0.003	0.200
All samples	/	/	/	0.322	/	/	/	/

*Note:* *p<0.05, **p<0.01, the remaining *p* > 0.05.

Estimates of gene flow calculated with the Migrate‐n program indicated the possibility of genetic exchange among the isolated island population (*T. b. modesta*), the two mainland‐isolated populations (*T. b. tonquinia* and *T. b. gaoligongensis*), and the combined mainland population (Table 4). Asymmetrical gene flow was clearly observed. The estimate of historical gene flow from the island population (*T. b. modesta*) to the combined mainland population was low (1.67 migrants per generation). In contrast, gene flow into the island population was substantially higher, with estimates from the combined mainland population, *T. b. tonquinia*, and *T. b. gaoligongensis* being 6.58, 9.69, and 16.79, respectively. Similarly, emigration from *T. b. tonquinia* and *T. b. gaoligongensis* to the combined mainland population was low (1.56 and 1.62, respectively), while immigration into these populations from others was moderate to high (e.g., 5.42 from the mainland to *T. b. tonquinia*, and 10.13 from *T. b. gaoligongensis* to *T. b. tonquinia*). Notably, gene flow among the three geographically structured populations (*T. b. modesta*, *T. b. tonquinia*, and *T. b. gaoligongensis*) was generally higher than their respective emigration rates to the continuous mainland population.

**TABLE 4:** Gene flow of *Tupaia belangeri*.

Parameter	Mean
*T. b. modesta* → Combined lineage	1.67
*T. b. tonquinia* → Combined lineage	1.56
*T. b. gaoligongensis* → Combined lineage	1.62
Combined lineage → *T. b. modesta*	6.58
*T. b. tonquinia* → *T. b. modesta*	9.69
*T. b. gaoligongensis* → *T. b. modesta*	16.79
Combined lineage → *T. b. tonquinia*	5.42
*T. b. modesta* → *T. b. tonquinia*	6.18
*T. b. gaoligongensis* → *T. b. tonquinia*	10.13
Combined lineage → *T. b. gaoligongensis*	3.88
*T. b. modesta* → *T. b. gaoligongensis*	8.00
*T. b. tonquinia* → *T. b. gaoligongensis*	9.96

## 4 Discussion

We reconstructed the phylogenetic relationships, population structure, population history, and historical gene flow of *T. belangeri* using complete mitogenome data from 63 individuals from 12 populations in China (Figure 1A). Our analysis reveals a clear mitochondrial genetic structure, reflecting long‐term isolation between major evolutionary lineages formed by geological events, landscape heterogeneity, and population history.

### 4.1 Deep Mitochondrial Structure and Subspecies Delimitation

Phylogenetic analyses based on whole mitogenomes and concatenated PCGs consistently recovered four deeply divergent and well‐supported mitochondrial clades (Figure 1C). Three of these clades correspond to geographically restricted subspecies (*T. b. tonquinia*, *T. b. modesta*, and *T. b. gaoligongensis*), whereas individuals traditionally assigned to *T. b. chinensis*, *T. b. yunalis*, and *T. b. yaoshanensis* clustered into a single widespread mitochondrial lineage. The strong genetic differentiation among the four clades is supported by large pairwise genetic distances (0.093–0.118) and very high FST values (0.792–0.948) (Table 1), with AMOVA indicating that most mitochondrial variation is distributed among populations rather than within populations. These results challenge the classical subspecies classification based on morphology (Wang 1987) but are consistent with previous molecular studies using *COI* and *Mc1r* (Fu, He, et al. 2018; Fu, Wang, et al. 2018). Simplified genomic datasets recovered similar genetic structure among populations and supported a single major lineage encompassing *T. b. chinensis*, *T. b. yunalis*, and *T. b. yaoshanensis* (Ren et al. 2023). It is worth noting that three published sequences on the tree (NC_002521, AF217811, and AJ421453) constitute a strongly supported, distinct clade that is separate from the combined lineage (Figure 1C), but unfortunately their geographical origins are unclear. Moreover, Roberts et al. (2011) detected substantial genetic differentiation among *T. belangeri* from Vietnam, Myanmar, and Cambodia using mitochondrial *12S* and *16S rRNA* sequences, supporting deep divergence within the species. Thus, further exploration of the population structure requires the addition of more samples from both domestic and international geographical sources.

The discrepancy between morphological classification and mitochondrial genetic patterns observed here may have several possible explanations. Recent divergence among the four mitochondrial clades may account for the insufficient accumulation of morphological differences to distinguish them as distinct lineages, even though genetic differentiation has reached a high level. This scenario is plausible because genetic divergence typically precedes morphological divergence, as molecular changes accumulate more rapidly than phenotypic changes, especially in traits that are not under strong selection pressure. Phenotypic plasticity, the ability of organisms to exhibit different morphological phenotypes in response to environmental variations without changes in genotype, provides another key explanation for this discrepancy. As demonstrated in previous studies on vertebrates, morphological traits (e.g., skull shape in tigers) can be significantly influenced by environmental factors such as diet and habitat, leading to phenotypic variation that is not congruent with genetic lineage (Cooper et al. 2022). For *T. belangeri* , the traditionally recognized morphological differences among *T. b. chinensis*, *T. b. yunalis*, and *T. b. yaoshanensis* may reflect plastic responses to local environmental conditions rather than inherent genetic differences (Gao et al. 2017). Additionally, differential selection pressures acting on morphological versus genetic traits could drive the observed discordance: morphological traits may be under stabilizing selection to maintain adaptive phenotypes in specific habitats, while mitochondrial and nuclear genes evolve neutrally or under divergent selection related to other functional processes, resulting in independent evolutionary trajectories.

### 4.2 Miocene Divergence and Geological Drivers

Molecular dating places the origin of the major mitochondrial lineages of *T. belangeri* in the Miocene, with divergence times broadly consistent with previous interspecific and intrageneric estimates for Scandentia (Figure 1D) (Roberts et al. 2011; Kundu et al. 2022; Petrova et al. 2023). In particular, the divergence of *T. b. gaoligongensis* coincides temporally with the uplift and geomorphological reorganization of the Gaoligong Mountains (Clark et al. 2004). The deep mitochondrial separation of this subspecies, together with its distinct demographic history and high genetic diversity, suggests long‐term persistence in situ following orogenic isolation. By contrast, the split between *T. b. tonquinia* and *T. b. modesta* also dates to the Miocene and predates the final isolation of Hainan Island from the mainland at Pleistocene (Feng et al. 2012), implying that their divergence was initiated by historical geological and environmental changes on the mainland rather than by insular formation alone. Despite the ancient divergence among mitochondrial lineages, estimates of historical gene flow reveal asymmetric migration, particularly involving the *T. b. modesta* (Table 4). The substantially higher inferred immigration into *T. b. modesta* relative to emigration suggests episodic or recent connectivity from mainland populations, potentially facilitated by human‐mediated transport rather than natural over‐water dispersal, as tree shrews have limited dispersal ability across ocean barriers and do not exist on many nearby islands (Olson et al. 2005).

### 4.3 Demographic Histories Among Mitochondrial Lineages

Demographic analyses revealed pronounced differences in population history among the four mitochondrial lineages (Figure 2). The widespread mainland lineage exhibited clear signatures of population expansion, including a significantly negative *D* value for Tajima, insignificant mismatch statistics, and EBSP inferred growth after population decline (Table 3 and Figure 2A). The combination of high haplotype diversity and relatively low nucleotide diversity is characteristic of rapid population expansion after historical bottlenecks and has been widely documented in small mammals in southern China during Quaternary climatic fluctuations (Table 2) (Wan et al. 2021; Wang et al. 2021; Liu, Deng, et al. 2025). Although *T. b. tonquinia* and *T. b. modesta* occupy geographically restricted regions, both subspecies showed similar patterns of high haplotype diversity coupled with low nucleotide diversity, along with EBSP evidence of recent population growth (Table 3 and Figure 2B,C). These results suggest that even isolated populations may have experienced limited demographic expansion under favorable climatic conditions, albeit without reaching the genetic diversity levels observed in the continuous mainland population. In contrast, *T. b. gaoligongensis* displayed a distinct demographic trajectory. Its positive Tajima's *D* value, together with EBSP results indicating long‐term stability followed by a recent decline (Table 3 and Figure 2D), suggests persistence in a relatively stable refugial environment. The complex topography and environmental heterogeneity of the Gaoligong Mountains are known to buffer populations against large‐scale climatic oscillations and have been implicated as refugia for numerous vertebrate species (Lu et al. 2013; Li et al. 2024, 2025).

### 4.4 Genetic Diversity and Evolutionary Implications

Genetic diversity is a fundamental prerequisite for population persistence and evolutionary potential, as reduced diversity can limit environmental adaptability and increase extinction risk (Humphries et al. 1995; Ellegren and Galtier 2016). In this study, genetic diversity analysis showed that a more than fivefold variation in the genetic diversity among subspecies, which reached a high level of genetic variation (Table 2). Island population *T. b. modesta* exhibited the lowest nucleotide diversity (0.005), consistent with small effective population sizes and limited contemporary gene flow in geographically isolated populations (Ren et al. 2023). Similarly, *T. b. tonquinia*, restricted to mountainous terrain, also showed low diversity despite its continental distribution, indicating that geographical or ecological isolation, even in non‐island settings, can constrain gene flow and reduce genetic variability. By contrast, the combined mainland populations with relatively frequent gene flow exhibited high genetic diversity, in line with previous studies (Liu and Yao 2013; Hou et al. 2018). Notably, *T. b. gaoligongensis* exhibited the highest nucleotide diversity among all subspecies (0.025), consistent with long‐term persistence in a topographically complex region. High genetic diversity in this lineage parallels patterns observed in other taxa, such as takin (Yang et al. 2022), red panda (Hu et al. 2020), and gayal (Liu, Li, et al. 2025), from the Gaoligong Mountains and supports the view that this region functions as an important reservoir of genetic diversity. The findings of this study have important conservation implications, particularly for the subspecies with low genetic diversity (*T. b. modesta* and *T. b. tonquinia*). As the species is listed in CITES Appendix II, which aims to regulate international trade and promote conservation of species that may face threats if trade is not controlled, our results provide practical guidance for targeted conservation strategies. For *T. b. modesta*, the extremely low genetic diversity coupled with its island isolation suggests that it is highly vulnerable to environmental changes, disease outbreaks, and inbreeding depression; therefore, urgent conservation measures, such as habitat protection, population monitoring, and potentially assisted gene flow to increase genetic variability, should be implemented to reduce its extinction risk. Similarly, *T. b. tonquinia*, despite its continental distribution, suffers from low genetic diversity due to its restricted mountainous habitat, highlighting the need for conservation efforts focused on maintaining and restoring connectivity between its fragmented populations to facilitate natural gene flow.

## 5 Conclusions

In conclusion, our mitogenomic analyses reveal that *T. belangeri* exhibits pronounced genetic structure and deep divergence, with four well‐supported lineages. Three clades correspond to geographically restricted subspecies, while the widespread mainland lineage encompasses multiple nominal subspecies, challenging classical morphology‐based classifications. Divergence time estimates suggest Miocene origins, with major tectonic and geomorphological events driving lineage splits, and historical gene flow appears limited and asymmetric, particularly involving Hainan. Demographic histories differ among lineages, with the *T. b. gaoligongensis* lineage exhibiting long‐term stability and remaining populations showing varying degrees of expansion. Genetic diversity highlights the Gaoligong Mountains as a key reservoir, whereas island and montane populations maintain lower diversity. These findings highlight the combined effects of historical isolation, landscape features, and demographic processes in shaping mitochondrial genetic structure in small mammals across southern China.

## Author Contributions

**Tongtong Gu:** conceptualization (lead), funding acquisition (lead), software (lead), writing – original draft (equal), writing – review and editing (lead). **Lijuan Cao:** conceptualization (supporting), methodology (supporting), software (supporting), writing – original draft (equal). **Dongjie Liu:** conceptualization (supporting), methodology (supporting), software (supporting), writing – original draft (equal). **Chengyao Yang:** conceptualization (supporting), methodology (supporting), software (supporting), writing – original draft (supporting). **Zixin Lin:** conceptualization (supporting), methodology (supporting), software (supporting), writing – original draft (supporting). **Rongxiang Mu:** conceptualization (supporting), methodology (supporting), software (supporting), writing – original draft (supporting). **Wanlong Zhu:** funding acquisition (equal), project administration (equal), writing – review and editing (equal).

## Funding

This research was funded by the Yunnan Fundamental Research Projects (202601CI070016), National Natural Science Foundation of China (32500436 and 32560262), and College Students’ Innovative Entrepreneurial Training Plan Program (DC2025147).

## Conflicts of Interest

The authors declare no conflicts of interest.

## Supporting information


**Figure S1:** Phylogenetic tree inferred from 13 protein‐coding genes (PCGs) (ML). The three tree‐building methods produced similar topology and here we show the ML tree.
**Figure S2:** The convergence result between the two independent runs of molecular dating analysis. The closer all points are to the diagonal, the better the convergence.
**Table S1:** Samples and sequencing data information used in this study.

## Data Availability

The following information was supplied regarding the availability of DNA sequences: the complete mitogenomes of *Tupaia belangeri* are deposited in GenBank of NCBI under accession number PX872944–PX872999.
