# Supplementary material for: Mitogenomic Insights Into the Population Structure and Demographic History of Tree Shrews ( Tupaia belangeri ) in China
Source: Ecol Evol. 2026 Sep 10;16(9):e74338. doi: 10.1002/ece3.74338 (PMC13561631; doi:10.1002/ece3.74338)
Supplement: Supplementary file 1 [file ECE3-16-e74338-s001.docx]

**Supplementary Information for**

**Mitogenomic insights into the population structure and demographic history of tree shrews (*Tupaia belangeri*) in China**

Tongtong Gu^1,#,*^, Lijuan Cao^1,#^, Dongjie Liu^1,#^, Chengyao Yang^1^, Zixin Lin^1^, Rongxiang Mu^1^, Wanlong Zhu^1,*^

^1^ School of Life Sciences, Yunnan Normal University, Kunming, China

^#^Co-first author.

^*^Corresponding author: Tongtong Gu, Wanlong Zhu

E-mail: 240041@ynnu.edu.cn, zwl_8307@163.com

**Supplementary Information includes:**

Figs. S1-S2

Table S1

**Fig. S1 Phylogenetic tree inferred from 13 protein-coding genes (PCGs) (ML).** The three tree-building methods produced similar topology and here we show the ML tree.

**Fig. S2 The convergence result between the two independent runs of molecular dating analysis.** The closer all points are to the diagonal, the better the convergence.

**Table S1** **Samples and sequencing data information used in this study.**

| Taxon | Species | Accession number | References | Mean base coverage | Length |
| --- | --- | --- | --- | --- | --- |
| Scandentia |  |  |  |  |  |
|  | *T. belangeri* | PX872944 | This study | 2,262 | 16,747 |
|  | *T. belangeri* | PX872945 | This study | 471 | 16,741 |
|  | *T. belangeri* | PX872946 | This study | 1,564 | 16,750 |
|  | *T. belangeri* | PX872947 | This study | 2,117 | 16,743 |
|  | *T. belangeri* | PX872948 | This study | 1,416 | 16,848 |
|  | *T. belangeri* | PX872948 | This study | 1,685 | 16,776 |
|  | *T. belangeri* | PX872950 | This study | 1,691 | 16,761 |
|  | *T. belangeri* | PX872951 | This study | 826 | 16,760 |
|  | *T. belangeri* | PX872952 | This study | 1,925 | 16,750 |
|  | *T. belangeri* | PX872953 | This study | 1,294 | 16,758 |
|  | *T. belangeri* | PX872954 | This study | 1,382 | 16,743 |
|  | *T. belangeri* | PX872955 | This study | 1,230 | 16,755 |
|  | *T. belangeri* | PX872956 | This study | 576 | 16,757 |
|  | *T. belangeri* | PX872957 | This study | 2,015 | 16,779 |
|  | *T. belangeri* | PX872958 | This study | 2,119 | 16,760 |
|  | *T. belangeri* | PX872959 | This study | 2,348 | 16,787 |
|  | *T. belangeri* | PX872960 | This study | 1,553 | 16,762 |
|  | *T. belangeri* | PX872961 | This study | 1,612 | 16,764 |
|  | *T. belangeri* | PX872962 | This study | 525 | 16,749 |
|  | *T. belangeri* | PX872963 | This study | 1,819 | 16,750 |
|  | *T. belangeri* | PX872964 | This study | 1,620 | 16,761 |
|  | *T. belangeri* | PX872965 | This study | 1,263 | 16,764 |
|  | *T. belangeri* | PX872966 | This study | 1,767 | 16,744 |
|  | *T. belangeri* | PX872967 | This study | 1,171 | 16,754 |
|  | *T. belangeri* | PX872968 | This study | 1,863 | 16,746 |
|  | *T. belangeri* | PX872969 | This study | 1,240 | 16,764 |
|  | *T. belangeri* | PX872970 | This study | 320 | 16,744 |
|  | *T. belangeri* | PX872971 | This study | 1,102 | 16,774 |
|  | *T. belangeri* | PX872972 | This study | 1,485 | 16,752 |
|  | *T. belangeri* | PX872973 | This study | 1,778 | 16,766 |
|  | *T. belangeri* | PX872974 | This study | 897 | 16,756 |
|  | *T. belangeri* | PX872975 | This study | 982 | 16,770 |
|  | *T. belangeri* | PX872976 | This study | 929 | 16,749 |
|  | *T. belangeri* | PX872977 | This study | 1,352 | 16,763 |
|  | *T. belangeri* | PX872978 | This study | 1,152 | 16,744 |
|  | *T. belangeri* | PX872979 | This study | 1,153 | 16,819 |
|  | *T. belangeri* | PX872980 | This study | 1,190 | 16,769 |
|  | *T. belangeri* | PX872981 | This study | 1,410 | 16,777 |
|  | *T. belangeri* | PX872982 | This study | 1,864 | 16,748 |
|  | *T. belangeri* | PX872983 | This study | 2,069 | 16,336 |
|  | *T. belangeri* | PX872984 | This study | 2,346 | 16,750 |
|  | *T. belangeri* | PX872985 | This study | 714 | 16,752 |
|  | *T. belangeri* | PX872986 | This study | 1,134 | 16,751 |
|  | *T. belangeri* | PX872987 | This study | 1,285 | 16,753 |
|  | *T. belangeri* | PX872988 | This study | 1,621 | 16,765 |
|  | *T. belangeri* | PX872989 | This study | 4,098 | 16,758 |
|  | *T. belangeri* | PX872990 | This study | 1,958 | 16,751 |
|  | *T. belangeri* | PX872991 | This study | 1,713 | 16,751 |
|  | *T. belangeri* | PX872992 | This study | 1,467 | 16,755 |
|  | *T. belangeri* | PX872993 | This study | 1,313 | 16,760 |
|  | *T. belangeri* | PX872994 | This study | 1,252 | 16,778 |
|  | *T. belangeri* | PX872995 | This study | 2,347 | 16,830 |
|  | *T. belangeri* | PX872996 | This study | 1,806 | 16,770 |
|  | *T. belangeri* | PX872997 | This study | 2,072 | 16,752 |
|  | *T. belangeri* | PX872998 | This study | 2,004 | 16,749 |
|  | *T. belangeri* | PX872999 | This study | 563 | 16,746 |
|  | *T. belangeri* | NC_002521 | Petrova, et al., 2023 | / | 16,754 |
|  | *T. belangeri* | AF217811 | Schmitz, et al., 2000 | / | 16,754 |
|  | *T. belangeri* | JN800724 | Xu, et al., 2012 | / | 16,805 |
|  | *T. belangeri* | JN800723 | Xu, et al., 2012 | / | 16,758 |
|  | *T. belangeri* | JN800722 | Xu, et al., 2012 | / | 16,791 |
|  | *T. belangeri* | AJ421453 | Arnason, et al., 2002 | / | 16,745 |
|  | *T. belangeri* | OP210313 | Direct Submission | / | 16,777 |
|  | *T. nicobarica* | NC_065160 | Kundu, et al., 2022 | / | 17,164 |
|  | *T. splendidula* | NC_050994 | Kundu, et al., 2022 | / | 16,189 |
|  | *T. minor* | NC_050993 | Kundu, et al., 2022 | / | 16,187 |
|  | *T. tana* | NC_050992 | Kundu, et al., 2022 | / | 16,186 |
|  | *T. glis* | NC_068695 | Direct Submission | / | 16,756 |
|  | *T. montana* | NC_054191 | Kundu, et al., 2022 | / | 16,183 |
|  | *Dendrogale murina* | NC_072603 | Kundu, et al., 2022 | / | 16,850 |
|  | *Ptilocercus lowii* | / | https://github.com/ZaTaxon/Ptilocercus_lowii | / | 10,954 |
| Dermoptera |  |  |  |  |  |
|  | *Galeopterus variegatus* | NC_004031 | Arnason, et al., 2002 | / | 16,748 |
